# Phenotypic Characterization and Transcriptome Analysis of the Dwarf Mutant *zmbrd1* in Maize

**DOI:** 10.3390/genes16121410

**Published:** 2025-11-27

**Authors:** Li Qin, Yu Bao, Chunlei Du, Xiaolong Guo, Xiaoduo Lu, Fugui Xie

**Affiliations:** 1 Institute of Advanced Agricultural Technology, Qilu Normal University, Jinan 250200, China; qinli2020520@163.com (L.Q.); 20251178@qlnu.edu.cn (Y.B.); guoxiaolong@nwafu.edu.cn (X.G.); 2 School of Geography and Tourism, Qilu Normal University, Jinan 250200, China; 15275260785@163.com; 3 Lab of Molecular Breeding by Design in Maize Sanya Institute, Hainan Academy of Agricultural Sciences, Sanya 572000, China

**Keywords:** maize, ZmBRD1, brassinosteroid, RNA-seq

## Abstract

**Background:** Maize (*Zea mays* L.) is a vital global crop, and yield improvement through dwarfing breeding—inspired by the Green Revolution—holds promise for addressing food security challenges. Despite the identification of over 60 dwarf genes in maize, their genetic diversity remains limited. Brassinosteroids (BRs) are key phytohormones that regulate plant height, and mutations in BR-related genes often result in dwarf phenotypes. **Methods:** The *zmbrd1* mutant was generated via EMS mutagenesis in the B73 background. Phenotypic traits (plant height, root length) and histological features (e.g., mesocotyl cell length) were compared between mutant and wild-type plants. Transcriptome sequencing of leaves and root tips identified differentially expressed genes (DEGs), followed by GO and KEGG enrichment analyses. Key hormone-related genes were validated by means of qRT-PCR. **Results:** The *zmbrd1* mutant exhibited severe dwarfism and reduced root length, primarily due to inhibited longitudinal cell elongation in internodes. Transcriptome analysis revealed 1652 DEGs in leaves and 1450 DEGs in roots. Enriched pathways included BR biosynthesis, plant hormone signal transduction, and glutathione metabolism. In leaves, upregulated genes were linked to hormone signaling and chloroplast function, while downregulated genes involved oxidoreductase activity and stress response. In roots, DEGs were enriched in ethylene signaling, MAPK pathways, and plant–pathogen interaction, suggesting impaired defense responses. qRT-PCR confirmed dysregulation of hormone-related genes: GA biosynthesis genes were downregulated, whereas auxin-related genes were upregulated in leaves but downregulated in roots. **Conclusions:** The dwarf phenotype of *zmbrd1* stems from disrupted BR biosynthesis, leading to hormonal imbalance (particularly in GA and auxin pathways), oxidative stress, and suppressed cell elongation. Our results suggest that ZmBRD1 plays a key role in integrating aboveground and underground growth likely through modulating hormone crosstalk. This study elucidates BR-mediated height regulation and provides genetic resources for maize breeding.

## 1. Introduction

Maize (*Zea mays* L.) is the largest food crop globally and in China [1]. However, challenges such as population growth, decreasing arable land, increasing demands for bioenergy, and global climate change have severely constrained maize production development. One effective approach to mitigate this issue is to enhance maize yield. Plant height influences crop lodging resistance, photosynthetic efficiency, and harvest index and is closely related to the final crop yield [2]. As one of the most important agronomic traits in breeding, excessively tall plants are prone to lodging, leading to yield reduction. Dwarf plants exhibit strong lodging resistance and high light utilization efficiency within the population, thereby increasing per mu yield. Consequently, dwarf stature, upright leaf angle, and high yield have been long-standing goals for breeders for decades [3].

Since the 1960s, the widespread adoption of dwarf and semi-dwarf crops triggered the first “Green Revolution” in agricultural production, significantly increasing grain yields [4,5]. The earliest utilization of dwarf breeding in maize dates back to the 1950s with the *br2* gene, a recessive dwarf gene controlling shortened internode length in maize [6]. It is the most extensively studied and applied dwarf gene to date. Following the identification of the *br2* gene, domestic and international research has continuously deepened the exploration of new dwarf genes. Currently, over 60 dwarf genes have been identified in maize, with 35 mapped to chromosomes [7,8,9]. It has been reported that several dwarf monogenic traits in maize have been cloned, including *br2*, *d3*, *d5*, *na2*, *VP8*, *bv1*, *D8*, and *D9*. Among these, only d1 and na1 genes located on chromosome 3 have been cloned. Previous studies cloned the maize dwarf gene *d1* via map-based cloning, demonstrating that it encodes GA3-oxidase, a key enzyme regulating the final step of the gibberellin (GA) biosynthesis pathway [7]. Multani et al. (2003) confirmed that the maize dwarf gene *br2* encodes an MDR (Multidrug Resistance) protein, which regulates polar auxin transport, thereby affecting plant height [10]. Winkler (1995) successfully cloned the maize dwarf gene *d3* using Mu transposon tagging; further research showed that *d3* encodes a protein involved in the GA synthesis pathway, and phenotypic responses are induced by alterations in GA content [11]. The dominant dwarf gene *D8* in maize was successfully cloned; it is GA-insensitive and encodes a protein similar to a nuclear transcription factor involved in the GA signal transduction process [12]. Liu et al. (2020) utilized transgenic technology to overexpress the dwarfing gene *ZmDWF4*, which improved maize agronomic traits and significantly enhanced heterosis in hybrid combinations with transgenic plants [13]. However, the current genetic diversity of dwarf resources is limited, making them difficult to apply in maize breeding. Therefore, the identification of novel dwarf resources and the screening or cloning of new dwarf genes are of great significance for dwarfing breeding.

Maize dwarfism is primarily regulated by plant hormones. GA, auxin (IAA), and BR are the major hormones influencing plant height [14]. However, a single hormone alone cannot effectively promote or inhibit plant growth; the dwarfism observed in many plants is often the result of coordinated regulation by multiple hormones. Furthermore, numerous genes that promote cell elongation and cell regeneration also play crucial roles in modulating plant height [15,16]. BRs are essential steroidal hormones for plant growth and development, regulating numerous vital processes including root growth, stem elongation, leaf expansion, and stress resistance [17,18]. The *Brd1* gene, which encodes a C-6 oxidase catalyzing the final step in BR biosynthesis, leads to a significant reduction in plant height when mutated [19]. Similarly, the maize ZmBRI1a gene encodes a plasma membrane-localized, leucine-rich receptor kinase whose extracellular domain binds BRs to initiate signaling. Mutation of this gene impairs the BR signal transduction pathway, reducing cell division and inhibiting cell elongation, consequently leading to plant dwarfism [20]. Mutations in key BR biosynthetic genes *Na1* and *Na2* disrupt distinct steps, leading to dwarfism. The *Na1* mutation causes dwarfism by accumulating (24R)-24-methylcholest-4-en-3-one, a DET2 substrate, which disrupts downstream BR metabolism [21]. Conversely, the *Na2* mutation impairs the early step of converting 24-methylenecholesterol to campesterol, inhibiting BR synthesis and causing additional phenotypes like fewer tillers and altered leaves [22].

Based on this, the present study utilized an EMS-induced mutant of the key BR biosynthetic gene *ZmBRD1* and its wild-type B73 background as experimental materials. By integrating phenotypic characterization and transcriptome analysis to investigate the genetic mechanisms underlying *ZmBRD1* function, this research aims to elucidate novel regulatory pathways controlling plant height in maize. The findings are expected to provide a new theoretical foundation for the genetic improvement of maize and establish a crucial research basis for developing germplasm resources adaptable to diverse cultivation models.

## 2. Materials and Methods

### 2.1. Experimental Materials

We used maize inbred lines B73 and *zmbrd1* mutants (Zm00001d033180, EMS4-2d86b5). The *zmbrd1* mutant was purchased from maizeEMSDB (http://maizeems.qlnu.edu.cn/) (Jinan, Shandong, China) [23].

### 2.2. Identification of Genotype of Mutant Strains

Fresh leaves were collected from 20 field-grown *brd1* mutant plants at the pollen-shedding stage. Genomic DNA was extracted using the CTAB method. The mutation site of the *brd1* premature termination mutant was queried in the MEMD mutant database. Sequencing primers were designed to flank 500 bp upstream and downstream of the mutation site (Table S1). PCR amplification was performed using DNA from *brd1* plants as the template. The PCR reaction mixture included 1 μL of template DNA (1 μg/μL), 1 μL of each forward and reverse primer, 5 μL of 2× Phanta Flash Master Mix (Dye Plus; Vazyme Biotech Co., Ltd.) (Nanjing, China), and nuclease-free water to a final volume of 10 μL. The PCR protocol consisted of initial denaturation at 95 °C for 90 s, followed by 30 cycles of denaturation at 95 °C for 20 s, annealing at 60 °C for 20 s, and extension at 72 °C for 10 s, with a final extension at 72 °C for 5 min. The amplified PCR products were sent to Tsingke Biotechnology Co., Ltd. (Beijing, China) for sequencing.

### 2.3. Bioinformatics Analysis of ZmBRD1 Gene

The protein sequence of ZmBRD1 was submitted to the OrthoDB v12.1 database (https://www.orthodb.org/?gene=4577_0:0012b0, accessed on 20 November 2025) for homologous gene retrieval. OrthoDB employs a hierarchical orthology classification system based on conserved gene families across major evolutionary lineages. The search parameters were set to default thresholds to identify orthologs with high sequence similarity. Genes from closely related species (e.g., *Z. mays*, *Sorghum bicolor*, *Oryza sativa*, and other monocots) showing significant alignment scores (E-value < 1 × 10^−10^) were selected. The corresponding gene accession numbers and protein sequences of these orthologs were downloaded in FASTA format for further analysis. Export sequences and draw evolutionary trees using DNAMAN 9 software and MEGA 7 software.

### 2.4. Transcriptome Sequencing

Transcriptome sequencing was performed using leaves and root tips at the three-leaf-one-heart stage of maize B73 and the *brd1* mutant, with three biological replicates set up for each sample. The transcriptome sequencing process included total RNA extraction, assessment of RNA purity and integrity, library construction, library quality control, and sequencing, which were completed by Beijing TsingKe Company Ltd.

The raw sequencing reads were processed to obtain high-quality clean reads. Using the maize B73 genome as the reference genome, the clean reads were aligned to the reference genome using the STAR v2.7.11b software. After alignment, the mapped reads were assembled and quantified. Differential gene expression analysis was performed using DESeq v1.8.3, with the criteria of FDR < 0.05 and |log_2_FC| > 1 (where FC represents fold change) to identify differentially expressed genes (DEGs).

Gene Ontology (GO) functional enrichment analysis was conducted on the DEGs. Additionally, pathway enrichment analysis was performed using the Kyoto Encyclopedia of Genes and Genomes (KEGG) database. Based on the pathways defined in the KEGG database and using the reference genome as the background, the differentially expressed genes were mapped onto KEGG pathway diagrams. With the significance threshold set at *p* < 0.05, the clusterProfiler package in R v4.5.2 software was used for the enrichment analysis and visualization of the DEGs, and the ggplot2 v4.0.1 package was employed for graphing.

### 2.5. Observation of Tissue Paraffin Sections

Sections were prepared using the paraffin section method, as described by [24].

### 2.6. Gene Expression Level Analysis

The expression levels of genes were analyzed via quantitative real-time PCR (qRT-PCR) using the ChamQ Universal SYBR qPCR Master Mix (Vazyme, Nanjing, China). The primers used are listed in Table S1. The qRT-PCR reaction was performed in a 20 µL volume containing 10 µL of 2× AceQ qPCR SYBR Green Master Mix, 0.4 µL of each forward and reverse primer, 2 µL of cDNA template, and 7.2 µL of ddH_2_O. The thermal cycling protocol was as follows: initial denaturation at 95 °C for 5 min; 40 cycles of denaturation at 95 °C for 10 s, and annealing/extension at 60 °C for 30 s. The relative expression level of the target gene was calculated using the 2^−ΔΔCT^ method [25]. Data are presented as the mean ± SD from three independent biological replicates. The *Actin* gene was used as an internal control for normalization.

## 3. Results

### 3.1. Identification of ZmBRD1 Mutant

**Figure 1:**
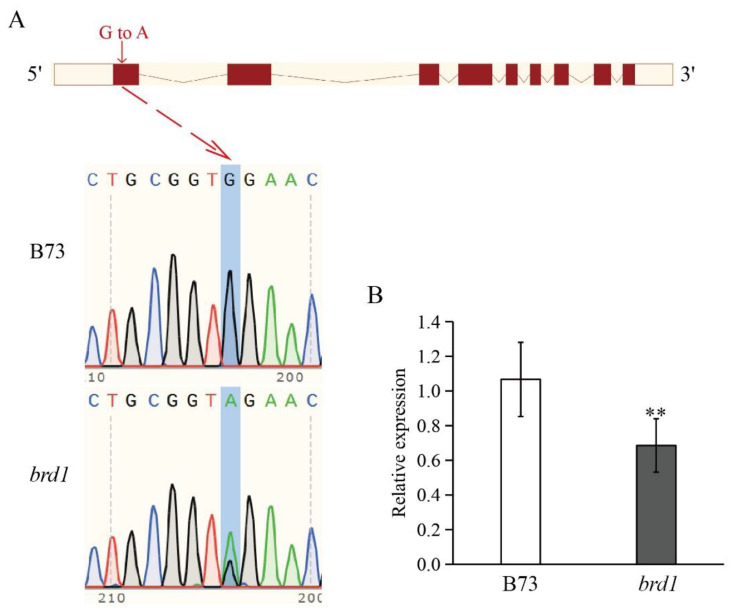
Identification of *zmbrd1* mutant. (**A**) *ZmBRD1* mutation location and *brd1* mutant sequencing results. In the schematic, CDS are denoted by red boxes, introns by lines, and UTRs by yellow boxes. (**B**) Expression levels of *ZmBRD1* in B73 and *brd1* mutant. Blue box indicates mutation location. ** represents significant difference compared with B73 at *p* < 0.05 level (*t*-test).

To investigate the function of the *ZmBRD1* gene, a single-base mutant of *ZmBRD1*, designated *brd1*, was obtained from a B73 maize mutant library. The mutation results in premature translation termination, where the 68th base in the CDS sequence is changed from G to A (Figure 1A, Table S2). The wild-type control used was B73. qRT-PCR analysis revealed that the expression level of the *ZmBRD1* gene in the mutant was significantly reduced compared to that in the wild-type (Figure 1B), indicating that this single-base mutation profoundly affects *ZmBRD1* gene expression.

### 3.2. Evolutionary Tree Analysis of ZmBRD1

**Figure 2:**
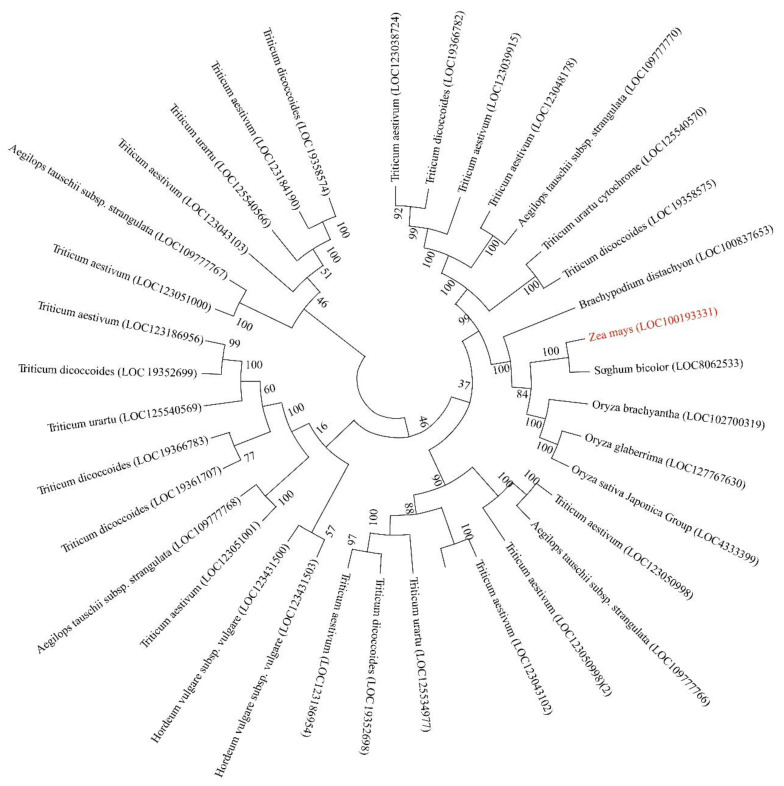
Analysis of the evolutionary tree of the maize ZmBRD1 family.

Protein BLAST v2.14.0 was employed to screen species encoding proteins with high homology to the ZmBRD1 family, including rice (*O. sativa*), wheat (*Triticum aestivum*), barley (*Hordeum vulgare*), and others. Phylogenetic analysis revealed that maize (*Z. mays* LOC100193331) and sorghum (*S. bicolor* LOC8062533) formed a clade with exceptionally high statistical support (e.g., bootstrap value ≥ 95%), indicating a robust evolutionary relationship between them (Figure 2). The clustering of ZmBRD1 with its sorghum ortholog in a short-branch clade suggests two key implications: (1) Evolutionary divergence: maize and sorghum likely diverged from a common ancestor approximately 12 million years ago, followed by independent evolution; (2) Functional conservation: the close phylogenetic relationship implies that BRD1 genes in both species may retain similar functions in regulating traits such as plant height and leaf angle. A notable observation from the phylogenetic tree is the expansion of BRD1 homologs in common wheat (*T. aestivum*), which harbors dozens of LOC identifiers. This proliferation strongly suggests large-scale gene duplication events in the wheat genome. As an allohexaploid, wheat possesses three sub genomes (A, B, and D) derived from whole-genome duplication events. The retention and diversification of multiple BRD1 copies across these sub genomes may underlie functional redundancy (where copies perform overlapping roles) or neofunctionalization (where copies acquire new functions. This genetic complexity potentially contributes to wheat’s elaborate architecture regulation and environmental adaptability. The abundance of BRD1 homologs provides a rich genetic foundation for modulating plant height and lodging resistance. Further investigation into the expression patterns, functional specificity, and synergistic roles of these copies will be crucial for advancing wheat breeding strategies.

### 3.3. The Impact of ZmBRD1 on Plant Architecture in Maize

**Figure 3:**
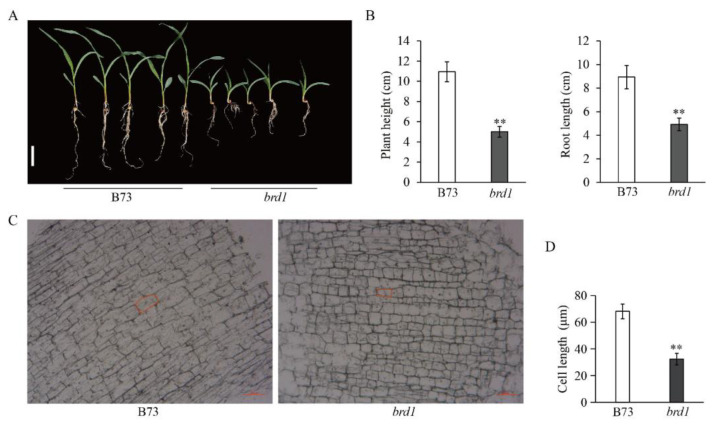
Effect of *ZmBRD1* on maize plant architecture. (**A**–**C**) Comparison of plant height and root length between wild-type and *brd1* mutant after 10 days of indoor growth. (**C**) Longitudinal paraffin sections of root from B73 (wild-type) and brd1 mutant seedlings. The red boxes highlight representative cells, visually demonstrating the severely inhibited longitudinal cell elongation in the brd1 mutant. (**D**) Average cell length of the two materials under the same field of view; Bars = 50 μm. ** represents significant difference compared with B73 at *p* < 0.01 level (*t*-test).

A comparative analysis of agronomic traits in 10-day-old seedlings revealed that the *brd1* mutant exhibited a significant reduction in both plant height and root length compared to the wild-type B73 (Figure 3A–C). This indicates that the single-base mutation in *ZmBRD1* exerts a significant positive effect on plant architecture traits such as plant height in maize. To elucidate the specific mechanisms underlying the dwarfism in brd1 mutants, a comparative histological analysis of mesocotyl cells was conducted between the dwarf mutant brd1 and the wild-type B73. Observations of longitudinal sections revealed a significant reduction in cell length in the brd1 mutant compared to the wild-type (Figure 3D). Statistical analysis of cell lengths measured from longitudinal sections under the same magnification further confirmed that the average cell length in brd1 was significantly shorter than that in B73 (Figure 3D). These findings demonstrate that the dwarf phenotype of the brd1 mutant is primarily attributed to impaired longitudinal cell elongation.

**Figure 4:**
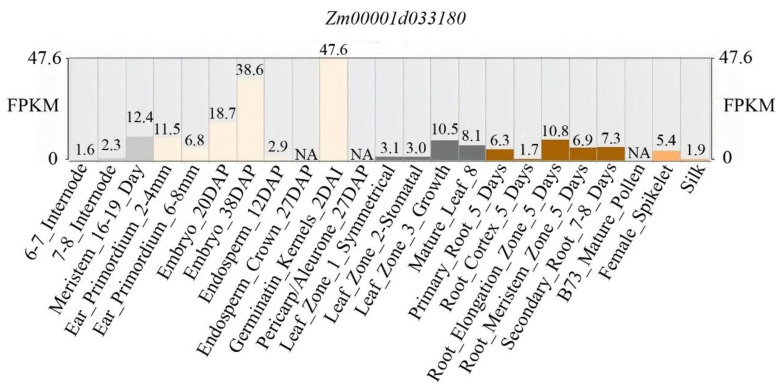
Spatiotemporal expression pattern of the *ZmBRD1* gene in different miaze Tissues.

Based on the MaizeGDB database, the expression profile of *ZmBRD1* across various tissues and developmental stages was analyzed. The results revealed a distinct tissue-specific expression pattern for *ZmBRD1* (Figure 4). Notably, the highest expression level (47.6 FPKM) was observed in Germination_Kernels_2DAI (kernels at 2 days after imbibition), suggesting its potential critical role in seed germination activation. During embryo development, the expression of *ZmBRD1* increased from 18.7 FPKM at 20 days after embryogenesis to 38.6 FPKM at 36 days, indicating its sustained involvement in embryo morphogenesis and maturation regulation. In root tissues, *ZmBRD1* expression was significantly higher in the root elongation zone (10.8 FPKM) compared to other regions, such as the root apical meristem (6.9 FPKM), implying a specific role in regulating root cell elongation. Furthermore, *ZmBRD1* exhibited dynamic regulation during development: its expression decreased from 11.5 FPKM in young spikelet primordia (2–4 mm) to 6.8 FPKM as the primordia grew to 6–8 mm, highlighting its active function in early spike organ development. *ZmBRD1* also showed regional specificity in leaves, with higher expression in Leaf_Zone_3_Growth (10.5 FPKM) compared to Leaf_Zone_1/2 (approximately 3 FPKM), suggesting preferential activity in meristematically active regions of the leaf. In contrast, expression in stem internodes was consistently low (below 2.3 FPKM), indicating a limited role during the mid-to-late vegetative growth stages. In summary, *ZmBRD1* appears to function as a core regulator governing germination–embryogenesis–root elongation processes.

### 3.4. Transcriptome Analysis of zmbrd1 Mutant Leaf Tissue

**Figure 5:**
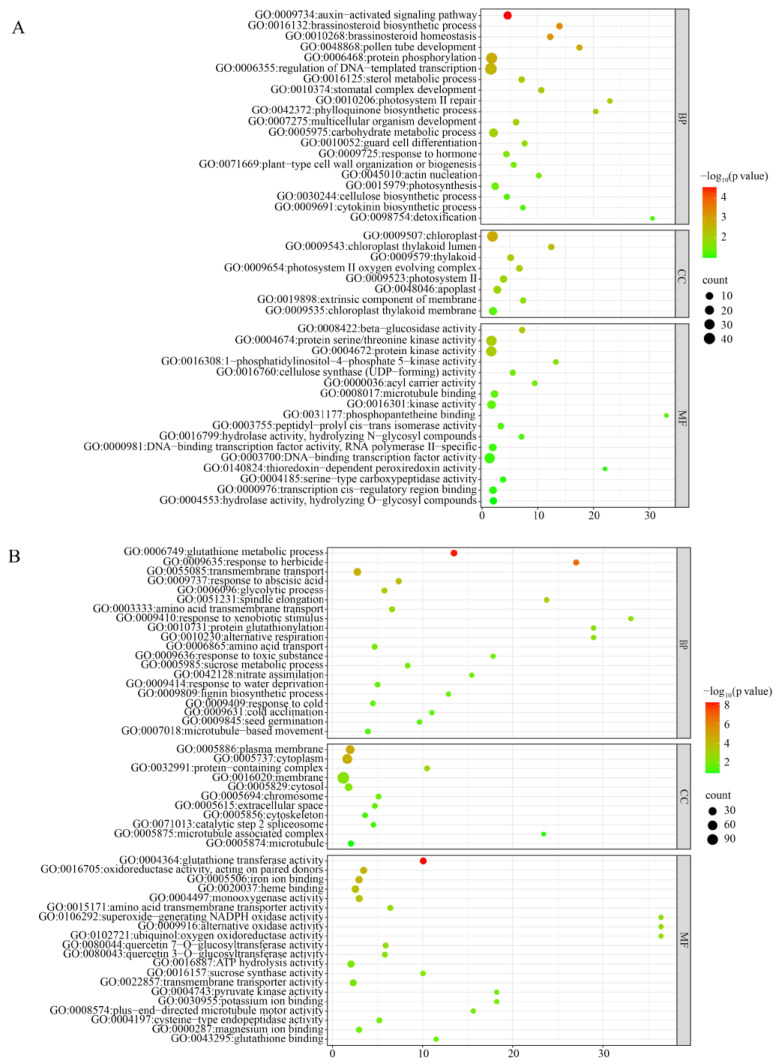
GO enrichment analysis of differentially expressed genes in leaf tissues of wild-type and mutant plants: (**A**) upregulated DEGs; (**B**) downregulated DEGs.

To investigate whether the mutation in the *ZmBRD1* gene affects the expression of other genes, transcriptome sequencing was performed using RNA extracted from leaf and root tip tissues of both the B73 wild-type and the *brd1* mutant (Table S3). Differentially expressed genes (DEGs) were identified using the thresholds of |log_2_FC| ≥ 1 and *p*-value < 0.05. In leaf samples, a total of 1652 DEGs were detected between the mutant and wild-type, comprising 927 upregulated and 725 downregulated genes. The 1652 DEGs were subjected to KEGG functional annotation and Gene Ontology (GO) enrichment analysis to elucidate their biological roles. In the *brd1* mutant leaves, the most significantly enriched BP (Biological Process Terms) for upregulated genes were closely associated with plant hormone signal transduction and synthesis (Figure 5A). Specifically, the auxin-activated signaling pathway (GO:0009734) and the brassinosteroid biosynthetic process (GO:0016132) were prominently enriched. Other enriched processes included photosynthesis (GO:0015979), response to abscisic acid (GO:0009737), and chloroplast organization (GO:0009658). These findings indicate that the *ZmBRD1* mutation broadly affects plant growth, development, and stress responses. CC (Cellular Component) enrichment analysis revealed that the upregulated genes in *brd1* leaves were most significantly localized to chloroplast-related structures. These included the chloroplast itself (GO:0009507), the thylakoid lumen (GO:0009543), the photosynthetic membrane (GO:0034357), and components of the photosystem (GO:0009521, GO:0009522). This strongly suggests that the gene expression changes induced by the *BRD1* mutation primarily occur within the chloroplast, directly impacting the site of photosynthesis. MF (Molecular Function) enrichment analysis showed that the most significantly enriched functions among the upregulated genes were related to protein kinase activity. This was particularly evident for protein serine/threonine kinase activity (GO:0004674) and the broader protein kinase activity (GO:0004672). Kinases are crucial components in signal transduction, which corroborates the enrichment of hormone signaling pathways found in the BP analysis and indicates a strong activation of signal transduction processes in the mutant.

Enrichment analysis of downregulated differentially expressed genes (DEGs) in *brd1* mutant leaves revealed significant associations with metabolic and stress-response processes. Key enriched terms included the glutathione metabolic process, indicating a decline in antioxidant and detoxification capacity in the mutant (Figure 5B). The response to herbicides was also prominently enriched, suggesting impaired defense mechanisms against environmental stressors. Additionally, downregulated genes were involved in diverse metabolic processes, reflecting broad disruptions in energy metabolism and biosynthetic functions. CC enrichment analysis demonstrated that downregulated genes were predominantly localized to the plasma membrane and cytoplasm. This localization implies that *BRD1* mutation may disrupt material transport and signal transduction pathways anchored at these sites. MF enrichment highlighted oxidoreductase activity as the most significantly enriched function among downregulated genes. Specific terms included oxidoreductase activity (GO:0016705) and glutathione transferase activity (GO:0004364). These results indicate that *BRD1* mutation leads to a marked downregulation of enzymes critical for maintaining cellular redox homeostasis. MF enrichment highlighted oxidoreductase activity as the most significantly enriched function among downregulated genes. Specific terms included oxidoreductase activity (GO:0016705) and glutathione transferase activity (GO:0004364). These results indicate that *BRD1* mutation leads to a marked downregulation of enzymes critical for maintaining cellular redox homeostasis.

**Figure 6:**
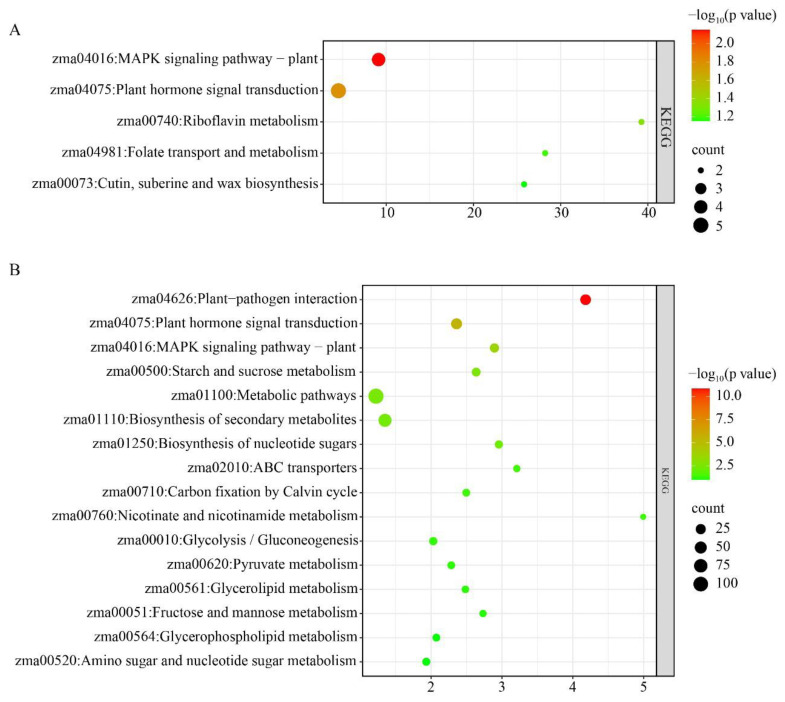
KEGG enrichment analysis of differentially expressed genes in leaf tissues of wild-type and mutant plants: (**A**) upregulated DEGs; (**B**) downregulated DEGs.

KEGG enrichment analysis demonstrated that metabolic pathways (zma01100), brassinosteroid biosynthesis (zma00905), and plant hormone signal transduction (zma04075) were highly significantly enriched among the upregulated differentially expressed genes (DEGs) in *brd1* mutant leaves (Figure 6A). Conversely, glutathione metabolism (zma00480) and metabolic pathways (zma01100) were highly significantly enriched among the downregulated DEGs (Figure 6B). These results indicate that normal BR signaling is essential for maintaining redox homeostasis in leaf cells. This oxidative stress state is likely a major factor contributing to the growth limitations observed in the mutant.

### 3.5. Transcriptome Analysis of Root Tissue of zmbrd1 Mutant

**Figure 7:**
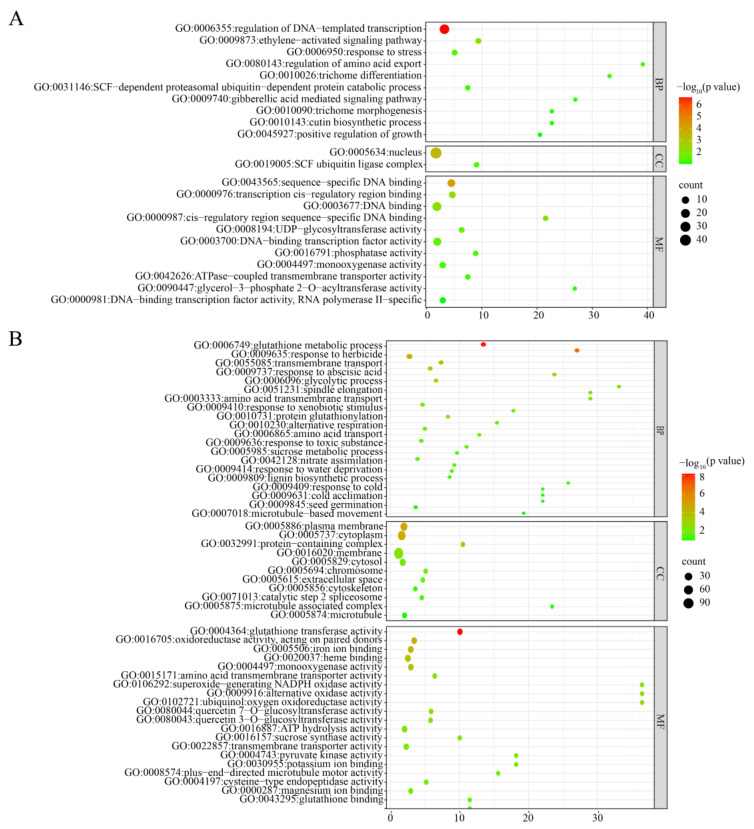
GO enrichment analysis of differentially expressed genes in root tissues of wild-type and mutant plants: (**A**) upregulated DEGs; (**B**) downregulated DEGs.

Comparative transcriptome sequencing of root tip tissues from the maize *brd1* mutant and its wild-type counterpart identified 1450 differentially expressed genes (DEGs), with 279 being upregulated and 1171 downregulated. Functional enrichment analysis of the upregulated DEGs was performed to elucidate the molecular consequences of the *brd1* mutation in roots. The most significantly enriched BP term was regulation of transcription, DNA-templated (GO:0006355), indicating a broad activation of transcriptional regulatory mechanisms in the mutant (Figure 7A). The ethylene-activated signaling pathway (GO:0009873) was also notably enriched, suggesting a role for ethylene signaling in the mutant’s response. CC enrichment analysis revealed that the upregulated genes were most significantly localized to the nucleus, consistent with their involvement in transcriptional regulation. The most significantly enriched MF terms were DNA-binding transcription factor activity (GO:0000976) and sequence-specific DNA binding (GO:0043565), highlighting the activation of transcriptional regulators.

Enrichment analysis of the downregulated differentially expressed genes (DEGs) in *brd1* mutant roots revealed that the most significantly enriched term was the protein phosphorylation process (GO:0006468) (Figure 7B). Additionally, the intracellular signal transduction process (GO:0035556) was also prominently enriched, indicating severe impairment of signal transduction mechanisms in the mutant. CC enrichment analysis demonstrated that the downregulated genes were most significantly localized to the plasma membrane and other membrane-associated structures. This suggests compromised membrane-related functions, which may affect critical processes such as signal perception and transmembrane transport. MF enrichment highlighted protein kinase activity (GO:0004672) and protein serine/threonine kinase activity (GO:0004674) as the most significantly enriched functions among the downregulated genes. Furthermore, terms such as calcium ion binding (GO:0005509) and calcium-dependent serine/threonine kinase activity (GO:0009931) were also notably enriched. These results collectively indicate a broad suppression of signaling transduction mechanisms in the roots of the *brd1* mutant.

**Figure 8:**
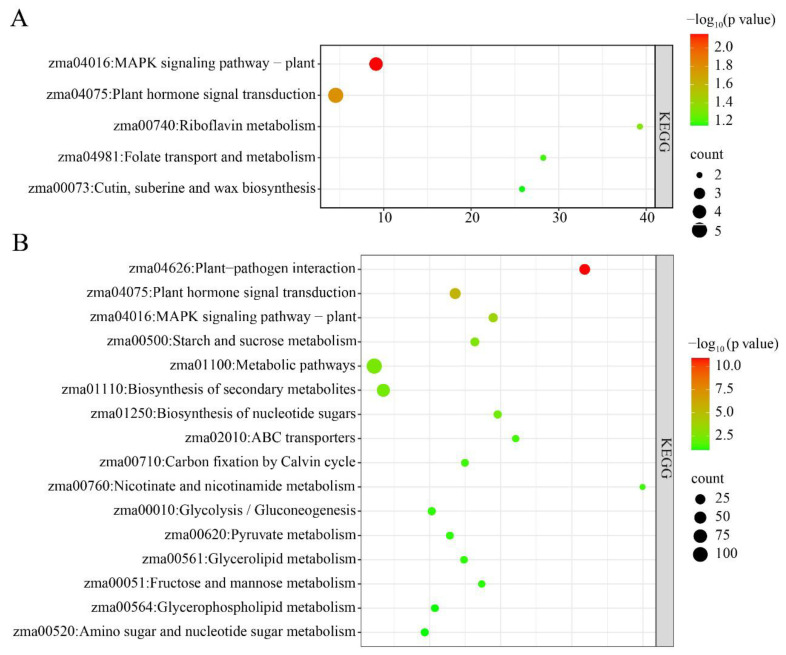
KEGG enrichment analysis of differentially expressed genes in root tissues of wild-type and mutant plants: (**A**) upregulated DEGs; (**B**) downregulated DEGs.

The simultaneous, strong activation of plant hormone signaling (e.g., auxin, ethylene, brassinosteroid and MAPK pathways among upregulated genes suggests a compensatory mechanism (Figure 8A). The plant may be attempting to rewire its signaling networks to maintain basic developmental processes and manage internal stress caused by the disruption in brassinosteroid homeostasis resulting from the *brd1* mutation. The highly significant enrichment of the Plant–Pathogen Interaction pathway among the downregulated DEGs is particularly critical (Figure 8B). This finding strongly suggests that the *brd1* mutation leads to a broad suppression of genes involved in recognizing pathogens and activating immune responses. Consequently, the mutant likely has weakened disease resistance and would be more vulnerable to infections from soil-borne pathogens.

### 3.6. qRT-PCR Validation

**Figure 9:**
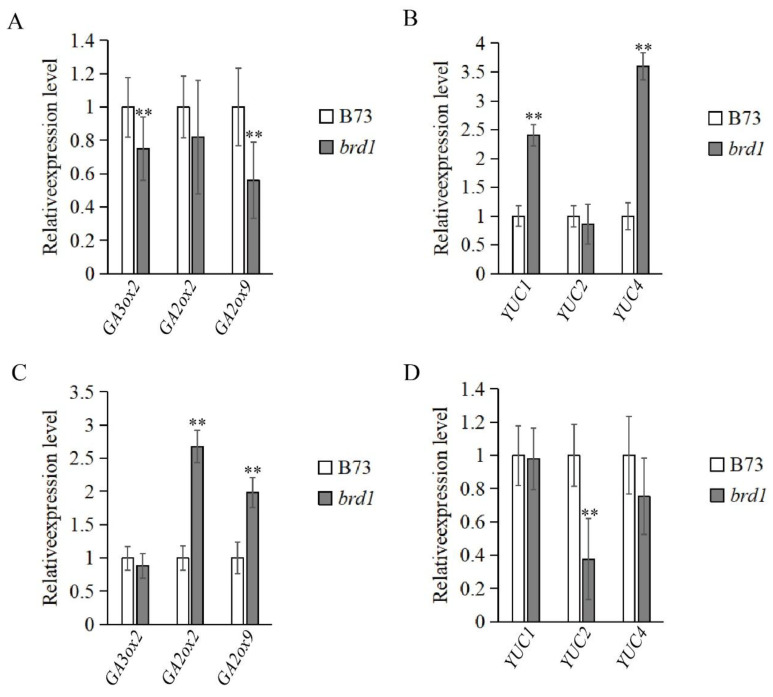
Results of quantitative Real-time PCR. (**A**,**B**) Quantitative analysis of selected auxin and gibberellin Pathway Genes expression in leaves of the *brd1* and wild-type. (**C**,**D**) Quantitative analysis of selected auxin and gibberellin Pathway Genes expression in roots of the *brd1* and wild-type. ** represents significant difference compared with B73 at *p* < 0.01 level (*t*-test). Data are presented as the mean ± SD from three independent biological replicates. The *Actin* gene was used as an internal control for normalization.

Based on the transcriptome analysis results, to better understand the mechanistic role of *BRD1* in regulating maize plant height, we investigated the expression levels of several representative genes related to auxin and gibberellin pathways in the brd1 mutant during plant height development (Figure 9). The results showed that in the leaves of the dwarf *brd1* material, gibberellin biosynthesis-related genes such as *GA3ox2* and *GA2ox9* were significantly downregulated, while *GA2ox2* showed no significant change. In contrast, auxin biosynthesis-related genes *YUC1* and *YUC4* were significantly upregulated in the leaves of *brd1*, whereas *YUC2* expression remained unchanged. Similar to the qRT-PCR results in leaves, gibberellin biosynthesis-related genes were significantly downregulated in the roots of *brd1*, while auxin biosynthesis-related genes were also downregulated in the roots of the mutant. These findings indicate that the loss of function of *BRD1* simultaneously disrupts the balance of both gibberellin and auxin pathways. *BRD1* may act as an upstream key factor in hormonal coordination, whose status directly affects the normal operation of multiple downstream hormone pathways.

## 4. Discussion

Plant height is a critical factor influencing crop yield, as it directly affects growth patterns, light utilization efficiency, lodging resistance, and adaptability to mechanical harvesting [26]. Since the Green Revolution, the strategic exploitation of dwarf mutants has significantly contributed to yield improvement in major crops [27]. Plant height is primarily determined by the number of internodes and the length of each internode [28]. Optimizing these traits through breeding can enhance photosynthetic efficiency, reduce lodging risks, and improve harvest efficiency, thereby collectively boosting yield. In monocot plants, internode elongation depends on the activity of the intercalary meristem located at the base of growing internodes, which drives both cell division and elongation [29,30]. For instance, the rice *D11* mutant exhibits dwarfism mainly due to shortened internodes rather than a reduction in internode number [31]. In contrast, the rice *htd3* mutant and maize *dnl2* mutant display dwarf phenotypes resulting from both fewer internodes and reduced internode length [32,33]. The present study demonstrates that the *brd1* mutant exhibits significantly reduced plant height compared to the wild type, accompanied by markedly smaller cells in the stem internodes. This suggests that the dwarfism may arise from impaired longitudinal cell development.

Furthermore, an alternative hypothesis that should be considered is that the observed phenotype might not solely result from a simple loss of BRD1 function but could also involve a negative dominance effect mediated by the expression of the truncated N-terminal peptide. While the nature of our mutation—a premature stop codon, the significantly reduced transcript level suggestive of Nonsense-Mediated mRNA Decay, and the phenotypic consistency with other known loss-of-function brd1 alleles—collectively supports a loss-of-function mechanism, the possibility of a dominant-negative effect cannot be excluded. If stably expressed, the truncated BRD1 protein fragment could potentially exert a dominant-negative effect by interfering with the function of the wild-type protein in heterozygotes or disrupting the activity of other related P450 proteins. Definitive discrimination between these two mechanisms will require future complementation tests with a true null allele, such as a complete gene deletion generated by CRISPR/Cas9. This intriguing possibility represents an important direction for future research.

Hormones play crucial roles in regulating plant growth and development, particularly plant height [34]. In barley, silencing the *HvGA2ox9* gene not only led to a significant increase in plant height but also promoted root growth [35]. In contrast, tomato plants overexpressing the *SlJAZ2* gene exhibited reduced plant height and internode length, decreased trichome density on stem internodes, and earlier flowering time [36]. In rice, mutations in the brassinosteroid-related gene *OsHFR131* resulted in plants exhibiting insensitivity to BRs, while the auxin response factor OsARF17 could bind to the promoter region of *HFR131* and positively regulate its expression, collectively modulating plant height [37]. In cucumber, the cytochrome P450 gene *CsCYP85A1* was identified as a putative candidate for the *super compact-1* (*scp-1*) mutation; the mutant exhibited extremely dwarfed stature with almost no internode elongation and a shrunken appearance, a phenotype that could be rescued through exogenous application of BR [38].

To further investigate the molecular mechanisms underlying the dwarf phenotype of the *brd1* mutant, an in-depth analysis of transcriptome data from its leaves and roots was conducted. GO and KEGG enrichment analyses revealed that in leaves, the majority of differentially expressed genes were significantly enriched in biological processes related to plant hormone signal transduction and metabolism. Given the critical regulatory roles of hormones in plant growth and development [39,40,41], it is hypothesized that in the leaves of the *brd1* mutant, the deficiency in a key step of BR biosynthesis leads to reduced levels of endogenous active BRs. This may trigger a compensatory feedback mechanism in which the cell attempts to upregulate biosynthetic genes. However, the attenuated BR signaling profoundly affects the synergistic network with other hormones, disrupting the growth-promoting hormonal environment [17,42]. More critically, it leads to the downregulation of antioxidant systems such as glutathione metabolism, rendering the cells vulnerable to oxidative stress [43]. Specifically, we observed a significant downregulation of ZmGST23, a tau-class GST gene known to be induced by diverse abiotic stresses including drought, salinity, and hormone treatments in maize. The compromised expression of this key detoxification enzyme likely impairs the mutant’s ability to scavenge reactive oxygen species (ROS) and manage oxidative damage, thereby providing a molecular link between the BR deficiency in zmbrd1 and its increased susceptibility to oxidative stress. Future work measuring ROS accumulation and validating the antioxidant capacity will be crucial to fully confirm this mechanism. The combined action of these two factors ultimately disrupts the normal growth and development program in the leaves of the *brd1* mutant, resulting in the associated phenotypic abnormalities. Furthermore, based on qRT-PCR results, the expression of several key GA biosynthetic genes was suppressed in the *brd1* mutant. This likely leads directly to a decrease in the levels of biologically active gibberellins in the plant. Gibberellins are crucial hormones promoting internode cell elongation [44]; their insufficiency is probably a primary cause of the shortened internodes and reduced plant height (i.e., dwarfism) observed in *brd1*.

The root system is responsible for absorbing water and minerals from the soil, while the shoot system produces organic compounds through photosynthesis [45]. A well-developed root system provides substantial water and nutrient support for greater plant height and more vigorous shoot growth; conversely, a tall canopy requires a robust root system for mechanical anchorage to prevent lodging [46,47]. In this study, the *brd1* mutant exhibited not only significantly reduced plant height but also markedly shorter root length compared to the wild type, suggesting that BRD1 may serve as a key hub coordinating the synergistic growth of aboveground and underground parts in maize. By regulating BR biosynthesis, it directly influences its own signaling pathway and indirectly affects other pathways, thereby simultaneously modulating these two important traits: plant height and root length. According to the root transcriptome results, differentially expressed genes in the roots of *brd1* were primarily enriched in key physiological processes such as transcriptional regulation, ethylene-activated signaling pathway, and MAPK signaling pathway. Therefore, we hypothesize that the loss of BRD1 function likely impairs BR biosynthesis, leading to significant inhibition of its downstream signaling pathway—which involves extensive protein phosphorylation—thereby directly hindering normal root development. In response to this severe internal dysfunction, root cells activate stress responses represented by the ethylene and MAPK pathways and initiate large-scale transcriptional reprogramming in an attempt to compensate and adapt [48,49]. However, these responses appear insufficient to restore normal growth, ultimately resulting in severely compromised root development.

In summary, this study investigated a newly identified maize dwarf mutant, *brd1*. Phenotypic characterization confirmed that its dwarfism is primarily attributed to a significant reduction in plant height caused by smaller cell size in the internodes. Transcriptome analysis further revealed that the majority of differentially expressed genes were significantly enriched in pathways related to plant hormones, suggesting that the dwarf phenotype of *brd1* is likely associated with mutations affecting genes involved in hormone biosynthesis or signaling. These findings not only deepen our understanding of the molecular mechanisms underlying plant dwarfism but also provide valuable genetic resources for breeding new maize varieties with ideal plant architecture.

## Data Availability

The raw sequencing data have been deposited in the National Genomics Data Center (NGDC), under the accession number PRJCA051445.

## Supplementary Materials

The following supporting information can be downloaded at https://www.mdpi.com/article/10.3390/genes16121410/s1. Table S1: Primers used in this study; Table S2: Comparison of characterized brd1 alleles in maize; Table S3: RNA-seq summary table.
